# Anticancer Activity of Lesbicoumestan in Jurkat Cells via Inhibition of Oxidative Stress-Mediated Apoptosis and MALT1 Protease

**DOI:** 10.3390/molecules26010185

**Published:** 2021-01-02

**Authors:** Joo-Eun Lee, Fang Bo, Nguyen Thi Thanh Thuy, Jaewoo Hong, Ji Shin Lee, Namki Cho, Hee Min Yoo

**Affiliations:** 1 Stem Cell Research Center, Korea Research Institute of Bioscience and Biotechnology (KRIBB), Daejeon 34141, Korea; jooeunlee83@gmail.com; 2 College of Pharmacy, Chonnam National University, Gwangju 61186, Korea; fangboplato@163.com (F.B.); thanhthuy287phar@gmail.com (N.T.T.T.); 3 Department of Physiology, Daegu Catholic University School of Medicine, Daegu 42472, Korea; jhong@cu.ac.kr; 4 Department of Pathology, Chonnam National University Medical School, Gwangju 61469, Korea; jshinlee@jnu.ac.kr; 5 Biometrology Group, Korea Research Institute of Standards and Science (KRISS), Daejeon 34113, Korea

**Keywords:** lesbicoumestan, MALT1/NF-κB, ROS, mitochondrial depolarization, 3D Jurkat cell

## Abstract

This study explores the potential anticancer effects of lesbicoumestan from *Lespedeza bicolor* against human leukemia cancer cells. Flow cytometry and fluorescence microscopy were used to investigate antiproliferative effects. The degradation of mucosa-associated lymphoid tissue lymphoma translocation protein 1 (MALT1) was evaluated using immunoprecipitation, Western blotting, and confocal microscopy. Apoptosis was investigated using three-dimensional (3D) Jurkat cell resistance models. Lesbicoumestan induced potent mitochondrial depolarization on the Jurkat cells via upregulated expression levels of mitochondrial reactive oxygen species. Furthermore, the underlying apoptotic mechanisms of lesbicoumestan through the MALT1/NF-κB pathway were comprehensively elucidated. The analysis showed that lesbicoumestan significantly induced MALT1 degradation, which led to the inhibition of the NF-κB pathway. In addition, molecular docking results illustrate how lesbicoumestan could effectively bind with MALT1 protease at the latter’s active pocket. Similar to traditional 2D cultures, apoptosis was markedly induced upon lesbicoumestan treatment in 3D Jurkat cell resistance models. Our data support the hypothesis that lesbicoumestan is a novel inhibitor of MALT1, as it exhibited potent antiapoptotic effects in Jurkat cells.

## 1. Introduction

Leukemia is a common cause of cancer-related deaths, and is the sixth most prominent cause in both men and women [1]. T-cell acute lymphoblastic leukemia is an aggressive malignancy that does not respond well to chemotherapy; according to one study, it is biologically dependent on the proteolytic activity of the mucosa-associated lymphoid tissue lymphoma translocation protein 1 (MALT1) protease [2]. In addition to being a paracaspase, MALT1 is a part of the CARD11–BCL10–MALT1 (CBM) complex, where it is the enzymatically active component. The CBM complex promotes lymphocyte proliferation and survival by cleaving the NF-κB inhibitors RelB, A20, and BCL-10, which activates NF-κB [2]. Various post-translational modifications, such as ubiquitination and phosphorylation, are vital in the regulation of CBM functions and signal transduction [2]. In addition to its protease activity, MALT1 promotes NF-κB activation via TRAF6 and the IKK complex by binding to BCL-10 and TRAF6 as a scaffold [3]. For these reasons, many involved in the development of chemotherapeutic treatments for leukemia view MALT1 as a promising potential drug target. Recently, MALT1 inhibition has been considered a therapeutic approach for inhibiting the NF-κB signaling pathway, which in turn reduces proliferation and induces apoptosis in T-cell leukemia cells [4]. In some studies, MI-2, mepazine, promazine, and thioridazine—which are all synthetic small-molecule showing inhibitory activity of MALT1—have been reported to inhibit activated B-cell-like diffuse large B-cell lymphoma [5]. Recently, new MALT1 inhibitory compounds derived from natural products, such as oxepinochromenones, have been discovered [6]. This manuscript reports our findings regarding the potential of lesbicoumestan from *Lespedeza bicolor* as a novel MALT1 inhibitor.

Researchers have managed to isolate various active compounds from *L. bicolor*, including alkaloids, flavonoids, sterols, and terpenoids with anti-inflammatory, antioxidative, cytotoxic, and blood-glucose-reducing effects [7,8]. We recently reported that isoflavonoids isolated from *L. bicolor* exhibit significant antileukemia potential [9]. Apoptosis (i.e., programmed cell death) is one of the most studied topics among cell biologists due to its key role in the pathogenesis of numerous diseases. Mitochondria are known to actively produce reactive oxygen species (ROS); ironically, they appear to be the primary victims of their own ROS production [10]. Further study revealed that cells can also be damaged through the generation of ROS. In response to mitochondrial damage, cells activate a complex set of signaling networks leading to the initiation of downstream signal cascades that control cell-cycle arrest, DNA repair, and apoptosis. Therefore, ROS-mediated oxidative damage plays an important role in apoptosis. We hypothesized that lesbicoumestan from *L. bicolor* could also exert apoptotic effects on human leukemia cancer cells via ROS-mediated oxidative damage.

Upon comparing the structural similarities of reported MALT1 inhibitors, we selected lesbicoumestan as a potential MALT1 inhibitor to investigate the potential mechanism of action underlying the traditional use of *L. bicolor*. To evaluate whether MALT1 was correlated with lesbicoumestan-induced cell death, MALT1 and NF-κB were analyzed using a Western blotting assay. Additionally, we employed molecular docking to further research the potential binding mode between lesbicoumestan and MALT1. In molecular docking, complementary values at binding sites of a macromolecular structure are scored based on a process in which small molecules are docked into the macromolecular structure [11]. Notably, we used a three-dimensional (3D) spheroid culture of Jurkat cells for drug screening, as it mimics the human tumor environment with respect to morphology, function, and drug resistance [12].

The objectives of this study can be summarized as follows: (1) evaluate the anticancer effects of lesbicoumestan on human leukemia cancer cells in 2D and 3D in vitro models; (2) explore the mechanism of action of lesbicoumestan-induced MALT1 inhibition on the proliferation and survival of Jurkat cells; and (3) identify the possible binding modes of lesbicoumestan to MALT1 protease.

## 2. Results

### 2.1. Lesbicoumestan-Induced Mitochondrial and Caspase-Dependent Apoptosis in Jurkat Cells

**Figure 1:**
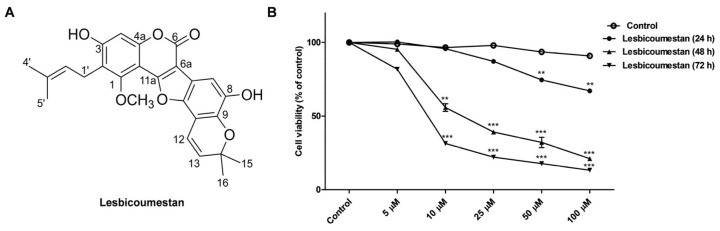
(**A**) Structure of lesbicoumestan. (**B**) Antiproliferative effects of lesbicoumestan on Jurkat cells (*n* = 3, ** *p* ≤ 0.01, *** *p* ≤ 0.001 vs. control group).

**Figure 2:**
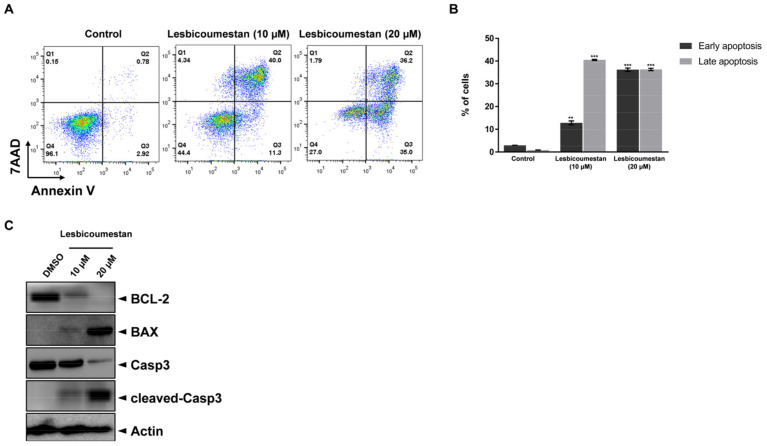
Lesbicoumestan-induced apoptosis. (**A**) Jurkat cells were either treated with lesbicoumestan or untreated for 48 h. Flow cytometry was subsequently employed to evaluate apoptosis. (**B**) Quantification results of early and late apoptotic cells (*n* = 3, ** *p* ≤ 0.01, *** *p* ≤ 0.001 vs. control group). (**C**) Whole-cell lysates from lesbicoumestan-treated cells were immunoblotted with antibodies specific for BCL-2, BAX, caspase-3 (Casp3), and cleaved caspase-3 (cleaved-Casp3).

**Figure 3:**
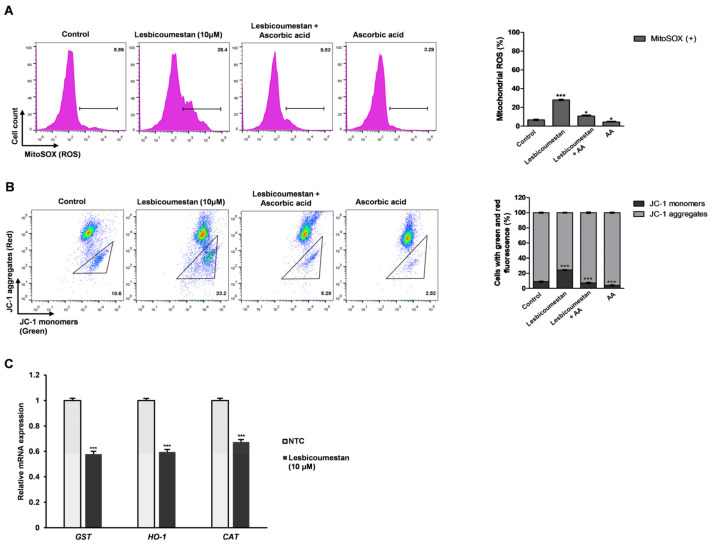
Lesbicoumestan-induced apoptosis via reactive oxygen species (ROS) generation, mitochondrial dysfunction, and reduced the mRNA expression level of antioxidant genes. (**A**) Jurkat cells were treated with lesbicoumestan and ascorbic acid (AA), and mitochondrial ROS was indicated by MitoSOX Red. Flow cytometry was employed to analyze the Jurkat cells. (**B**) Mitochondrial membrane potentials (MMPs) of Jurkat cells treated with lesbicoumestan, as measured via flow cytometry with JC-1 (5′,6,6′-tetrachloro-1,1′,3,3′-tetraethylbenzimidazolylcarbocyanine iodide) staining. Quantification of the JC-1 green and red fluorescence of the experiments of silencing or overexpressing lesbicoumestan and ascorbic acid in Jurkat cells (*n* = 3, * *p* ≤ 0.05, *** *p* ≤ 0.001 vs. control group). (**C**) Effect of lesbicoumestan on the mRNA expression levels of involved antioxidants in Jurkat cells. The qRT-PCR analysis results of *GST*, *HO-1*, and *CAT* mRNA levels in the control and in the Jurkat cells treated with 10 μM lesbicoumestan (*n* = 3, *** *p* ≤ 0.001) were compared.

The structure of lesbicoumestan is shown in Figure 1A. We examined lesbicoumestan-induced apoptosis by stimulating the generation of reactive oxygen species (ROS) as well as mitochondrial dysfunction. As shown by the results in Figure 1B, lesbicoumestan exhibited dramatic antiproliferative activity against leukemia cells. The cell viability results also show that lesbicoumestan only exhibited cytotoxic effects within short time exposures (24 h) if high doses were used (50 µM and 100 µM). On the other hand, lesbicoumestan treatment could lead to dramatic losses in cell viability over long exposure times (72 h), even if treated with low doses (10 µM and 20 µM). We further conducted an evaluation of lesbicoumestan-induced apoptosis with Jurkat cells. To determine the percentages of viable and apoptotic cells, the Jurkat cells were stained with annexin V and 7-amino-actinomycin D (7-AAD) dyes (Figure 2A,B). According to the results gathered after 48 h of lesbicoumestan treatment, there were significant increases in early and late apoptotic cell percentages. Moreover, Western blot analysis was conducted to determine B-cell lymphoma 2 (BCL-2), Bcl-2 associated X (BAX), caspase-3, and cleaved-caspase-3 expression levels and evaluate whether caspase activation is involved in lesbicoumestan-induced apoptosis. As shown in Figure 2C, treatment with lesbicoumestan remarkably reduced the expression levels of BCL-2 and caspase-3 and increased the expression levels of BAX and cleaved caspase-3 in Jurkat cells in a dose-dependent manner. Next, we determined the effect of lesbicoumestan in regulating mitochondrial ROS and mitochondrial membrane potential (MMP). As a result, the lesbicoumestan-treated Jurkat cells were analyzed with significantly increased mitochondrial ROS levels (Figure 3A) and decreased MMP (Figure 3B). We additionally observed that ascorbic acid (AA) reversed the lesbicoumestan-induced expression of ROS and restored MMP to a significant level. To investigate the molecular mechanism, we performed a real-time quantitative polymerase chain reaction (RT-qPCR) experiment using antioxidant-related genes. The RT-qPCR analysis showed that lesbicoumestan treatment noticeably decreased the mRNA expression level of antioxidant genes such as *GST*, *HO-1*, and *CAT* (Figure 3C). Therefore, the data indicate that lesbicoumestan downregulates antioxidants, which consequently leads to apoptosis followed by the accumulation of ROS. All these data suggest that lesbicoumestan induced intrinsic mitochondrial apoptosis in Jurkat cells by activating the ROS-dependent and caspase-dependent pathways.

### 2.2. Lesbicoumestan-Induced Degradation of MALT1

**Figure 4:**
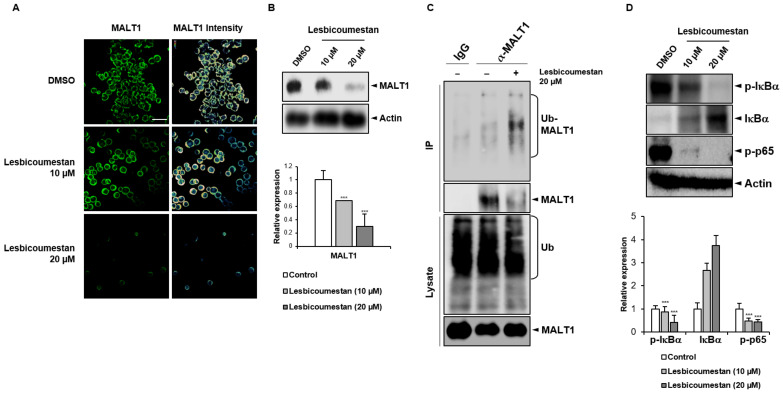
Lesbicoumestan-inhibited MALT1/NF-κB in Jurkat cells. (**A**) Immunofluorescence staining of MALT1 in Jurkat cells treated with 10 µM and 20 µM lesbicoumestan. (**B**) MALT1 expression following lesbicoumestan treatment of the cells (10 µM and 20 µM) as analyzed via Western blots. (**C**) SDS lysates of the indicated lines were subjected to control treatment or MALT1 antibody binding and protein A/G purification. Immunoblotting was performed to analyze purified proteins and total lysates. (**D**) Expression levels of p-IκBα, IκBα, and p-p65 after the cells were treated with lesbicoumestan (10 µM and 20 µM) as determined using whole-cell lysates. *n* = 3, *** *p* < 0.001 vs. control group.

To evaluate the ability of lesbicoumestan to target MATL1, we evaluated MALT1 expression via immunofluorescence following lesbicoumestan treatment (Figure 4A). According to the results, MATL1 expression decreased in Jurkat cells as a result of the lesbicoumestan treatment. Additionally, lesbicoumestan markedly downregulated the protein expression of MALT1 in a dose-dependent manner (Figure 4B). The ubiquitin-proteasome system is responsible for the degradation of target proteins via K48-linked polyubiquitination [13]. MALT1 is a major scaffold protein; its protease activity is involved in the signal transduction downstream of NF-κB [14]. To determine whether ubiquitin modified MALT1, an analysis was performed on MALT1 precipitates that were obtained following immunoprecipitation under denaturing conditions, which reduces nonspecifically bound proteins and prevents noncovalent protein interactions [15]. After treatment with lesbicoumestan and the proteasome inhibitor MG132, an anti-ubiquitin antibody was used to detect ubiquitinated MALT1 in the high-molecular-weight bands (Figure 4C). As shown in Figure 4D, lesbicoumestan strongly inhibited p65 phosphorylation. Furthermore, lesbicoumestan-treated cells exhibited a reduction in phosphorylated IκB and an increase in IκB protein. Our findings suggest that the inhibition of NF-κB is mediated by lesbicoumestan-induced MALT1 destabilization, which is a key upstream signal in NF-κB signaling.

### 2.3. Molecular Docking

**Figure 5:**
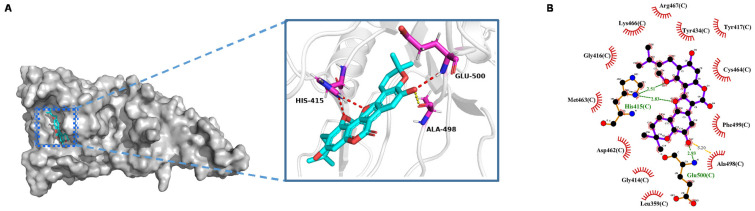
Calculated binding mode of lesbicoumestan with MALT1. (**A**) Overall and local structure of the binding mode. (**B**) 2D presentation of hydrophobic interactions between amino acid residues and lesbicoumestan.

To explore the possible binding mode of lesbicoumestan to MALT1, we obtained the crystal structure of MALT1 as a template (3UO8) [16] from the Protein Data Bank [17,18] and a docking study was conducted to rationalize the inhibitory activity of lesbicoumestan using Autodock4 [19]. For binding pocket estimation, we selected the pocket in which the origin ligand posed, which was previously reported as a known paracaspase domain of MALT1 [16,20]. By comparing lesbicoumestan to the reported MALT1 inhibitor β-lapachone, we found that both were structurally similar molecules and had a partially closed scaffold. Based on the reported structure–activity relationship of β-lapachone [21], the reservation of a pyran ring instead of an open-ring configuration is extremely important: it grants β-lapachone more favorable physicochemical properties and allows it to exhibit better activity on cell lines. In addition, several amino acid residues, including His415, Glu500, and Cys464, are believed to contribute to the key interactions of the paracaspase domain of MALT1 [17,21]. Figure 5A shows the best conformation of lesbicoumestan, which resulted in a low binding energy of −9.39 kcal/mol. As expected, the pyran part and its two methyl groups completely descended deep inside the binding pocket. A strong hydrogen bond formed between the oxygen atom at the C8 position and the main chain amino group of the Glu500 residue. In addition, the aforementioned oxygen atom was also near the Ala498 residue, separated by a distance of 3.2 Å. This is denoted by the yellow dashed line in Figure 5A, and could probably lead to an extra hydrogen bond. However, potential bonding interactions between lesbicoumestan and Cys464 were not observed. We assumed that the lack of a substituent at the C7 position, which is in contrast to β-lapachone, caused lesbicoumestan to move away from the Cys464. Based on this, we believe the phenolic hydroxyl group at the C8 position may play an extremely important role in generating a binding conformation of lesbicoumestan without help from Cys464. In addition, the oxygen atom adjacent to C11a also formed a hydrogen bond with the amino hydrogen of the His415 residue, which in turn formed an extra hydrogen bond with another oxygen atom at the C3 position. Moreover, according to the 2D presentation of the binding mode shown in Figure 5B, the butenyl side chain was located in the wide groove, forming favorable hydrophobic interactions with Gly416, Lys466, Arg467, Tyr434, and Tyr417. Lesbicoumestan could also hydrophobically interact with numerous other amino acid residues, including Asp462, Phe499, Ala498, Gly414, and Leu359.

### 2.4. Sensitivity of Lesbicoumestan on 3D Jurkat Cell Models

**Figure 6:**
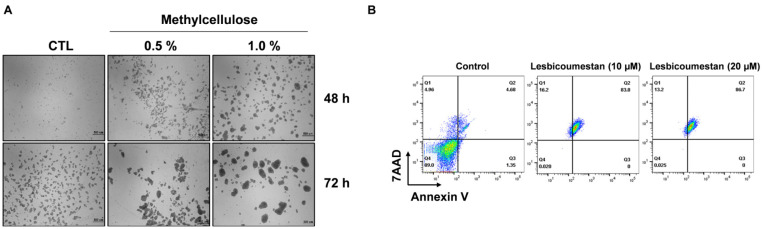
Effect of lesbicoumestan on a 3D Jurkat cell resistance model. (**A**) Representative images of methylcellulose-mediated spheroid formation on Jurkat cells. Cells were seeded in ultra-low-attachment 6-well flat bottom plates and treated with methylcellulose with concentrations of 0.5–1.0% for 48–72 h. Scale bar, 500 μm (*n* = 3 for each experiment). (**B**) Jurkat cells treated with the indicated concentrations of lesbicoumestan. Flow cytometry was employed to assess the cellular apoptosis of the 3D Jurkat cells 48 h post-treatment (*n* = 3).

Three-dimensional spheroid culture models represent in vivo cancer environments and can be used to study cell survival and drug resistance at the molecular mechanism level [12]. Three-dimensional Jurkat spheroid models were generated as described in our previous reports [22,23]. The cells were treated with 0.5% and 1.0% methylcellulose for 48 h and 72 h (Figure 6A). After 72 h, the Jurkat spheroids were treated with lesbicoumestan and subsequently stained with annexin V/7-AAF to evaluate apoptosis. As a result, the induction of apoptosis was determined to be dependent on the dose of lesbicoumestan (Figure 6B). The results suggest that lesbicoumestan induced significant cell death in the 3D cancer-resistant model.

## 3. Discussion

In this study, we observed that lesbicoumestan exhibited strong cytotoxic effects on Jurkat cells in vitro, which included mitochondrial depolarization, cell apoptosis, ROS induction, and the inhibition of tumor growth. Moreover, lesbicoumestan was shown to exert its effects via the MALT1/NF-κB signaling pathway.

We investigated the mechanism of action of lesbicoumestan on Jurkat T cells. An antiproliferation assay was performed to investigate the cellular activity of lesbicoumestan. A previous study reported that paracaspase MALT1 was indispensable for T-cell activation and proliferation, and that it also mediated T-cell antigen receptor-induced signaling to the transcription factor NF-κB [4]. Therefore, MALT1-mediated NF-κB activation can be diminished by inhibiting MALT1 proteolytic activity, which is a potential therapeutic approach for the treatment of leukemia. Our analysis also showed that the intensity of immunofluorescence staining for MALT1 was substantially reduced by lesbicoumestan in a dose-dependent manner. According to our Western blotting analysis results, lesbicoumestan interfered with inducible MALT1 activity in Jurkat cells (Figure 4B). In addition, following the immunoprecipitation of MALT1 from the Jurkat lysates, we observed that lesbicoumestan-treated MALT1 was conjugated by K48-linked poly Ub chains, leading to MALT1 degradation and the subsequent downregulation of phospho-IκBα and phospho-p65 expression levels; this result suggests that MALT1 stability is critical in delivering the signal to NF-κB cell activation in leukemia (Figure 4C,D). Lastly, our molecular docking results demonstrated that lesbicoumestan could effectively bind with the active pocket of MALT1 via broad hydrophobic interactions and favorable hydrogen-bonding interactions formed by His415 and Glu500 residues (Figure 5). This result provides a reference for the development of MALT1 inhibitors in addition to the related structural transformations and modifications of natural products.

Three-dimensional cultures have steadily developed into a powerful tool in the fields of cancer research and drug screening [24]. Three-dimensional cell culture models such as spheroids closely mimic the actual 3D environment of human tumors; these can be used for the investigation of anticancer agents [25,26]. However, drug resistance is one of the most significant obstructions in the treatment of hematological malignancies such as leukemia [27,28]. A recent study using Jurkat cells showed that 3D architecture is important for imparting chemoresistance [13]: cells that are sensitive to a particular drug in 2D cultures could potentially be resistant in 3D cultures. According to our analysis, apoptosis was markedly induced by lesbicoumestan treatment in a 3D resistance model in a similar manner to that seen in traditional 2D cultures (Figure 6). These results support the potential of lesbicoumestan as an apoptosis inducer in leukemia resistance models.

## 4. Materials and Methods

### 4.1. Plant Material, Extraction, and Isolation

*L. bicolor* roots were collected and lesbicoumestan was extracted as described in our previous publication [9].

### 4.2. Cell Viability Assay

Human Jurkat cell lines were obtained from the American Type Culture Collection (ATCC) and were cultured according to standard mammalian tissue culture protocols. Cell viability was evaluated using the MTS assay 48 h after treatment with lesbicoumestan, as described in our previous publication [29].

### 4.3. Apoptosis Assay

An apoptosis detection kit with PE annexin V and 7-AAD (BioLegend, San Diego, CA, USA) was used to analyze the Jurkat cells to determine apoptotic cell percentages. Once the cells were stained with PE annexin V and 7-AAD, a flow cytometer (BD FACSVerse, BD Biosciences, San Diego, CA, USA) was used to classify the cells as early (7-AAD-/annexin V+) or late (7-AAD+/annexin V+) apoptotic cells. The cell percentages were acquired using FlowJo software (Version 10, BD Biosciences). The detailed method followed the manufacturer’s instructions, as described in our previous publication [29].

### 4.4. Mitochondrial Membrane Potential (MMP) and ROS Assay

The cells were separately treated with MitoSOX Red mitochondrial superoxide indicator (Thermo Fisher Scientific, Waltham, MA, USA) and MitoProbe JC-1 (5′,6,6′-tetrachloro-1,1′,3,3′-tetraethylbenzimidazolylcarbocyanine iodide, Thermo Fisher Scientific) for 20 min at 37 °C to measure mitochondrial ROS levels and MMP, respectively. Both measurements were conducted using a flow cytometer (BD FACSVerse, BD Biosciences, San Diego, CA, USA). FACS buffer (1% fetal bovine serum in PBS) was used for washing and resuspension.

### 4.5. Western Blotting

Cell lysates were prepared in RIPA buffer and 1× protease inhibitor cocktail (Roche Applied Bioscience, Penzberg, Germany). The proteins were separated via sodium dodecyl sulfate-polyacrylamide gel electrophoresis (SDS-PAGE) on Mini-PROTEAN precast gels using a running buffer (Bio-Rad, Hercules, CA, USA) and were then transferred to PVDF membranes (0.45 μM). After being blocked with 5% nonfat milk, the membranes were incubated with the following primary antibodies according to the manufacturer’s recommendations: anti-cleaved BAX, BCL-2, caspase-3, phosphorylated I-kappa-B-alpha (p-IκBα), I-kappa-B-alpha (IκBα), phosphorylated p65 (p-p65), p65, actin (Santa Cruz Biotechnology, Inc., Santa Cruz, CA, USA), anti-MALT1, K48-linkage-specific polyubiquitin, and cleaved caspase-3 (Cell Signaling Technology, Danvers, MA, USA). The membranes were subsequently incubated with HRP-conjugated secondary antibodies (Jackson Laboratory, Bar Harbor, ME, USA). To scan and analyze the membranes, an ImageQuant LAS 4000 mini imager (Fujifilm, Tokyo, Japan) and an image analysis program (Multi Gauge Ver. 3.0, Fujifilm) were used.

### 4.6. RT-qPCR

Total RNA was extracted from cells treated with lesbicoumestan for 48 h using the RNeasy mini kit (Qiagen, Hilden, Germany). According to the manufacturer’s instructions, each PCR reaction was performed using the Maxima SYBR Green/ROX qPCR Master Mix (Thermo Fisher Scientific, Waltham, MA, USA). The RT-qPCR analysis was also performed on a StepOne Real-Time PCR system (Thermo Fisher Scientific, Waltham, MA, USA).

### 4.7. Ubiquitination of MALT1

To analyze MALT1 ubiquitination, the cells were treated with DMSO or lesbicoumestan (20 μM) and lysed by boiling in 150 mM Tris-HCl (pH 8.0), 5% SDS, and 30% glycerol. The cell lysates were diluted tenfold with RIPA buffer (Thermo Fisher Scientific) containing a protease inhibitor cocktail and 5 mM NEM. The cleared lysates underwent immunoprecipitation overnight with an anti-MALT1 antibody (Cell Signaling Technology). Following incubation with Dynabeads Protein A/G magnetic beads (Thermo Fisher Scientific) for 1 h, the magnetic beads were collected using a magnet. These beads were washed with RIPA buffer and boiled in SDS sampling buffer. The proteins were separated via SDS-PAGE and were then subjected to immunoblot analysis with MALT1 and K48-linkage-specific polyubiquitin antibody (Cell Signaling Technology).

### 4.8. Confocal Imaging

Cells treated with lesbicoumestan for 24 h were fixed in 4% paraformaldehyde. The fixed cells were blocked with 5% BSA and permeabilized with 0.05% Triton X-100 in PBS. MALT1 (Cell Signaling Technology) and donkey anti-rabbit Alexa Fluor 488 (Thermo Fisher Scientific) were used as the primary and secondary antibodies, respectively. Both antibodies had concentrations of 1:200. Binding was performed for 2 h at 4 °C, after which Hoechst 33342 (Thermo Fisher Scientific) was used to stain the DNA. Images were acquired using a confocal microscope (LSM800, Oberkochen, Germany) with Zen blue edition software (Zeiss, Oberkochen, Germany).

### 4.9. Generation of Spheroids

Three-dimensional spheroid tumor models were generated by seeding 10,000 cells/well; a final concentration of 0.5–1.0% methylcellulose was added to the cell suspension in ultra-low-attachment 6-well flat-bottom plates (Corning). The required amount of methylcellulose was titrated and determined accordingly. The plate was incubated at 37 °C in a 5% CO_2_ incubator for 48–72 h. Spheroids, which served as in vivo tumor-resistance models, were formed following the aggregation and tight clumping of cells. The spheroids were cultured for 1 day, 3 days, and 7 days under standard culture conditions.

### 4.10. Molecular Docking

ChemOffice (ChemOffice 15.0, Cambridge, MA, USA) was used to construct the 3D structure of the ligand and perform energy minimization. Following a structural optimization process, the lesbicoumestan molecule in pdbqt format was prepared for docking by merging non-polar hydrogen atoms, detecting rotatable bonds, and adding Gasteiger charges. The crystal structure of MALT1 was retrieved from the Protein Data Bank (code no. 3UO8). The molecular docking of lesbicoumestan to the crystal structure of human MALT1 was carried out using Autodock4 (Autodock4, Scripps Research Institute, San Diego, CA, USA) and visualized using PyMOL (PyMOL 2.4, New York, NY, USA) and LigPlot (LigPlot1.4, European Bioinformatics Institute, Cambridge, UK) [30]. Based on related molecular docking research [21] and the reported crystal construction of MALT1 with a small-molecule inhibitor, the grid box size was set as 96 Å, 96 Å, and 84 Å for x, y, and z, respectively. The spacing between the grid points was 0.375 Å. The grid center was set at −4.236 Å, −12.003 Å, and 3.972 Å for x, y, and z, respectively. The Lamarckian genetic algorithm (LGA) was implemented to search for the best conformers, and all docking processes were performed with the default parameters of AutoDock4. The best conformation was chosen based on binding energy and the hydrogen bond sites. The results were visualized using PyMOL and LigPlot [30], and hydrogen bonds and hydrophobic interactions were annotated.

### 4.11. Statistical Analysis

Statistical analysis was performed using GraphPad Prism (GraphPad Software, Inc., version 7, La Jolla, CA, USA), and the values are presented as the means ± SD. The data were further analyzed using the Student’s *t*-test, and a *p*-value < 0.05 was considered statistically significant.

## Data Availability

The data presented in this study are available in the article.

## Sample Availability

Samples of the compounds are available from the authors.
